# The Prevalence of Mental Distress and Social Support among University Students in Jordan: A Cross-Sectional Study

**DOI:** 10.3390/ijerph182111622

**Published:** 2021-11-05

**Authors:** Sawsan M. A. Abuhamdah, Abdallah Y. Naser, Ghada Mohammad Abdelwahab, Ahmad AlQatawneh

**Affiliations:** 1 Department of Pharmaceutical Sciences, Abu Dhabi Campus, College of Pharmacy, Al Ain University, Al Ain 64141, United Arab Emirates; 2 Department of Biopharmaceutics and Clinical Pharmacy, Faculty of Pharmacy, The University of Jordan, Amman 11942, Jordan; 3 Department of Applied Pharmaceutical Sciences and Clinical Pharmacy, Faculty of Pharmacy, Isra University, Amman 11622, Jordan; abdallah.naser@iu.edu.jo (A.Y.N.); ghada.abdelwahab@iu.edu.jo (G.M.A.); 4 ACDIMA Center for Bioequivalence and Pharmaceutical Studies, Department of Clinical Research, Amman 11190, Jordan; ahmad.y.qatawneh@gmail.com

**Keywords:** mental distress, social support, mental health, university students

## Abstract

Objectives: To examine the prevalence of mental distress among university students in Jordan. Methods: An online cross-sectional study using a self-administered questionnaire was conducted between 12th of June and the 4th of August 2021 in Jordan to measure student mental stress using Self-Reporting Questionnaire-20 (SRQ-20). Results: A total of 1063 university students participated in the study. One-third of the participating students reported that they had a history of COVID-19 infection. More than half of the participating university students (65.7%) were found to have mental distress (measured symptomatically by the SRQ-20 with a score of eight or more). The average mental distress score was 9.8 (SD: 5.5) out of 20. Female students, students from non-medical colleges, students in their last years of study, students with chronic diseases and those with low income were associated with high levels of mental distress (*p* < 0.05). With regards to social support, a moderate level of social support was received from three sources: persons considered as significant others, family members, and friends. The average social support score for the participating university students was 41.9 (SD: 10.3) out of 60 (equivalent to 69.8%). Conclusions: Mental distress is prevalent among university students in Jordan. There is a need for evidence-based governmental strategies and interventions that provide social support at universities such as self-help measures and professional mental health services as part of student health services that would be helpful to reduce the burden of mental distress of students and promote the mission of the integration of mental health in all university policies.

## 1. Introduction

One of the main problems that affect societies is mental distress, which includes anxiety, depression, sleeping problems (insomnia), and fatigue [1,2]. Different studies have emphasized that university students are considered to be at a higher risk (and higher level) of developing mental distress compared to the general public [1,2,3,4]. Studies in Australia have explored mental distress among medical students and found that there was a significantly higher incidence when compared to the general public [4,5]. Similarly, comparable findings were reported in different countries in Asia and Africa [3,6,7,8,9,10,11,12,13,14]. Previous studies conducted in Saudi Arabia (at Jizan University) and in Tanzania (in Dar Es Salaam) reported similar findings that 71.9% and 70.0% of the students have experienced mental distress, respectively [15,16]. A recent study was conducted in Germany on university students to examine their mental health problems and reported that 20.0% of them have signs of anxiety syndromes [17]. Another study in India explored the prevalence of psychological distress and associated coping strategies among healthcare profession students [18]. This study found that 27.7% and 9.7% of the students experienced anxiety and stress, respectively, during the pandemic [18].

Mental distress has multidimensional negative impacts on university students, including anxiety and depression, impaired cognitive functioning, poor academic performance, learning disabilities and substance abuse and is negatively correlated with physical activity [7,19,20,21]. Consequently, the presence of mental distress increases the probability of developing other mental health problems. Several contributing factors were proven to be associated with developing mental distress among university students, including female gender, loneliness and not having close friends, a lack of religious belief, low income, an overload of university duties and exam stress and a lack of interest in the field of study [1,5,10,16].

Due to the COVID-19 pandemic, which began in December 2019, multiple studies in the Middle East region (Jordan, Saudi Arabia, and Kuwait) have explored the social and mental burden of the COVID-19 pandemic on different sectors of the society, including university students, healthcare professionals and the general population [11,12,13,14,20,22]. A recent study in Saudi Arabia by Mohammed et al. assessed the psychological conditions among university students during the pandemic and found that 26.9% had anxiety symptoms and 22.4% had stress symptoms [23]. Additionally, they found that students who reported having a friend or relative infected with or that had died of COVID-19 and not getting emotional support from their family, university and society have a higher risk of having psychological problems [23]. Another study by El-Monshed et al. in Egypt that examined the psychosocial effects and coping strategies of university students during the COVID-19 pandemic reported that 47.1% and 40.5% of the students had anxiety and stress [24].

All studies agree that the pandemic has exerted a considerable amount of extra stress on individuals, resulting in a higher risk of developing anxiety, depression and other forms of mental illnesses, particularly among university students [11,12,13,14,20,22,25]. Due to the continuous financial and educational challenges that have been caused by the pandemic, mental health issues among university students may have intensified with time with the duration of the pandemic. To the best of our knowledge, there are no studies that have explored mental distress among university students in Jordan during the COVID-19 pandemic. Previous studies that explored mental distress among university students were mainly conducted in Africa and before the pandemic [1,7,8]. This study aims to explore the prevalence of mental distress among students in Jordan and its associated factors. Additionally, we examined the perceived social support from the students’ perspective.

## 2. Methods

### 2.1. Study Design

An online cross-sectional study using a self-administered questionnaire was conducted between 12th of June and the 4th of August 2021 in Jordan.

### 2.2. Sampling Strategy

We used convenience sampling techniques to recruit the study participants. University students from any level of study who were willing to participate in the study formed the study population. The inclusion criteria were (1) university students aged 18 years and above and (2) students studying at Jordanian university. Students who met these inclusion criteria were invited to participate in the study. These inclusion criteria were highlighted in the invitation letter of the survey. We used social media platforms such as WhatsApp, Twitter and Facebook to invite university students to participate in the study. The study aims and objectives were clearly explained at the beginning of the survey. Students were encouraged to participate in the study to assess the prevalence of mental distress and the perceived social support, which would help in identifying high-risk groups and provide recommendations for intervention to decrease the psychological burden of the pandemic.

### 2.3. Questionnaire Tool

Two previously validated assessment scales were used in this study [26,27]. Mental distress was measured using a self-reporting questionnaire scale (SRQ-20). This 20-item scale was originally developed by the World Health Organization (WHO) to indicate mental distress [1,28]. It was previously validated in different low and middle-income countries and was concluded to be highly specific and sensitive [28,29,30,31,32]. In our study, university students were asked if they experienced mental distress symptoms (measured symptomatically by the SRQ-20) within the past one month. The highest possible score for the SRQ-20 is 20. Those with a score of eight or more were considered to have mental distress [1,8]: the higher the score, the greater the level of mental distress. This scale was examined previously regarding its face, content and criterion validity. Additionally, its sensitivity, specificity and construct validity were demonstrated in different languages [28].

The second assessment scale was the social support questionnaire (SSQ). This scale was used to measure the perceived social support from the students’ perspective. This is a 12-item scale with responses ranging from 1 (strongly agree) to 5 (strongly disagree), with the highest score reflecting the highest level of social support [33]. The SSQ is sub-divided into three sub-scales forming three sources of social support, which are (1) significant other (a special person other than family members and friends who forms a source of social support), (2) family and (3) friends. Each sub-scale is measured with four items. The total possible score for the SSQ is 60, with a maximum score of 20 for each sub-scale. Previous studies indicated that the SSQ scale is psychometrically sound in diverse subject samples with strong factorial validity, good internal reliability, construct validity and test–retest reliability [27,33,34,35,36].

We examined the reliability of the two assessment scales using Cronbach’s alpha. The Cronbach’s alpha measures for the two assessment scales (SRQ-20 and SSQ) were 0.880 and 0.913, respectively, reflecting good to excellent internal consistency.

### 2.4. Sample Size

Using a confidence interval of 95%, a standard deviation of 0.5 and a margin of error of 5%, the required sample size was 385 participants.

### 2.5. Statistical Analysis

Data were analyzed using Statistical Package for Social Science (SPSS) software, version 27 (IBM Corp, Armonk, NY, USA). Continuous data were reported as mean ± standard deviation (SD). Categorical variables were reported as frequencies and percentages. An independent samples *t*-test and one-way analysis of variance (ANOVA) were used to compare the mean mental distress scores and mean social support scores between different demographic groups. Fisher’s least significance difference (LSD) post hoc test was used to identify the source of significant variation within each group.

Binary logistic regression analysis was used to explore predictors of mental distress among university students. The assumption of the statistical analyses (logistic regression) including the absence of multicollinearity among independent variables was checked.

A multiple linear regression analysis was used to explore the association between the social support score and mental distress score. Mental distress was defined as a total score of eight and above on the SRQ-20. A confidence interval of 95% (*p* < 0.05) was applied to represent the statistical significance of the results, and the level of significance was predetermined as 5%.

## 3. Results

A total of 1063 university students participated in the study. Around 71.0% of them were aged 18–23 years, and the rest of the sample were aged above 24 years. The majority of them (70.8%) were females, with males amounting to 29.2%. More than half of the study sample were studying at medical colleges (68.9%).

Around half of the study sample (48.9%) comprised students from first year to third year. Postgraduate students comprised 11.9% of the study sample.

The vast majority (90.0%) or respondents reported that they were single. Married students comprised 8.9% of the study sample. More than half of respondents (66.0%) reported that their income was 500 JD and below. Only 6.2% of the students reported that they had a history of chronic disease. One-third of the participating students (36.3%) reported that they had a history of COVID-19 infection (Table 1).

### 3.1. Mental Distress and Social Support for University Students

More than half of the participating university students (65.7%) were found to have mental distress (measured symptomatically by the SRQ-20 with a score of eight or more). The average mental distress score was 9.8 (SD: 5.5) out of 20. With regards to social support, a moderate level of social support was received from three sources: persons considered as significant others, family members and friends. The average social support score for the participating university students was 41.9 (SD: 10.3) out of 60. The social support score was similar for the three sub-scales, with an average score of 13.9 (SD: 4.4), 14.8 (SD: 3.9), and 13.2 (SD: 4.2) for significant others, family and friends, respectively (Table 2).

### 3.2. Mental Distress and Social Support for University Students Stratified by Demographic Characteristics

Table 3 shows the mean mental distress and social support score stratified by the demographic characteristics of university students. A higher mental distress score was observed among younger students, females, students of non-medical colleges, students in their first year of study, single students, low-income students (500 JD and below) and high-income students (1500 JD and above) and those who reported a history of chronic diseases (*p* < 0.05).

A higher social support score was observed among older students (30 years and over), students of medical colleges, students in their final year of study, students who were married or divorced, middle-income students (500 to 1000 JD) and those who reported a history of COVID-19 infection (Table 3). The LSD test confirmed that “age group 18–20 years, fourth and fifth year students, single students and those with an income of 500–1000 JD” were the main source of significant variation in mental distress score between groups. Besides, the results confirmed that “age groups 21–23 years and 30 years and over, fourth and fifth year students, single students and those with an income of 500–1000 JD” were the main source of significant variation in the social support score between groups.

Multiple linear regression analysis showed that a higher social support score was significantly associated with a lower mental distress score (*p* < 0.001).

### 3.3. Predictors of Mental Distress among University Students

Binary logistic regression analysis was used to identify predictors of mental distress among university students. Females, students of non-medical colleges, second and third year students, those who reported a history of chronic disease and those with a history of COVID-19 infection showed a higher risk of developing mental distress compared to others (*p* < 0.05) (Table 4).

## 4. Discussion

This study aimed to determine the prevalence of mental distress and social support for university students in Jordan. About 65.7% of the participating university students were found to have mental distress. Female gender, studying at non-medical colleges, being in the second and third years of study and having a chronic disease and COVID-19 infection history were found to be factors that increased the risk of developing mental distress (*p* < 0.05).

The prevalence of mental distress among university students in our study was higher than that of Chinese university students (41.1%) and students in Tanzania (14%) [26,37]. This significant difference may be attributed to the difference in the timing of the studies, where an earlier time is associated with a lower level of uncertainty among the study population and, ultimately, a lower level of mental distress and psychological burden. One of the main factors that increased the level of mental distress and psychological burden is the emergence of multiple mutations of SARS-CoV-2 genome [38]. Some of these variants showed non-response to COVID-19 vaccines [38]. Other contributing variables could be cultural differences and different response measures implemented by different countries in response to the pandemic.

Social support was found to be structurally and functionally associated with better mental health in college students [39]. The level of social support in our study was similar among the three sub-scales (with an average score of 13.9 (SD: 4.4), 14.8 (SD: 3.9), and 13.2 (SD: 4.2) for significant others, family and friends, respectively). University students received a moderate level of social support (41.9 (SD: 10.3) out of 60 (equivalent to 69.8%)). This suggests that having support from family members and friends reduces the stress associated with the change in the university environment in terms of the nature of education and the sudden transfer from the traditional learning model to remote learning [26]. In this study, a higher social support score was observed among older students (30 years and over), married and divorced students and those who reported a history of COVID-19 infection. This could be because these groups of participants have better connections and support from their family and friends compared to others. Students at medical college, those in their final years of study and middle-income students (500 to 1000 JD) were also observed to have greater social support. A recent study by Naser et al. in Jordan explored the impact of the COVID-19 pandemic on social relationships among more than 4000 people [13]. The study by Naser et al. reported that 31.6% of the participants were affected negatively in terms of their social relationships and communication with others by the COVID-19 pandemic. Additionally, the demographic characteristics contributed to the variation in being affected socially by the pandemic; for instance, participants who were aged 36–45 were positively affected in terms of their social relationships during the COVID-19 pandemic compared to others.

Being female was found to increase the level of mental distress, depression and anxiety, especially during the recent pandemic [11,12,13,14,20,40]. A higher mental distress score was observed among females in our study as well. This had been previously attributed to different variables including socioeconomic factors (such as abuse, education and income), biological sex differences, education and diet [41]. Another contributing factors could be the increased frequency of hormonal fluctuations in women compared with men [42,43]. A higher level of mental distress was also observed among younger students, students in their first year of study and single students. This was not consistent with a previous study by Ma, Z. et al. (2020), in which it was found that freshmen tended to have less academic pressure and worry less about the future [43]. Consistent with another study by Xiong P. et al. (2020), we found that students of non-medical colleges reported a higher prevalence of mental distress than medical students [44], and a higher level of distress was found among those who reported a history of chronic diseases, and, according to Louvardi, M. et al. (2020) [45], a higher level of mental distress was experienced by low-income students (500 JD and below) and high-income students (1500 JD and above). Previous studies showed that levels of stress decrease with age as the older population has a lower level of knowledge about possible complications of disease, and they have greater faith and submission to mortality [46], which could explain why younger students, first year students and single students had a higher level of mental distress compared to others. On the other hand, the elderly population is more likely to be affected by stressors and adapt to new situations [47,48,49].

Social support efforts and programs should be intensified and directed towards high-risk populations, including females, students of non-medical colleges, students in their last years of study, those who have reported a history of chronic disease and those with a history of COVID-19 infection. Social support should be multidimensional (emotional, instrumental and informational) and should be provided by relatives or friends and the society as a whole. Multidimensional social support is an important protective measure that decreases the probability and intensity of psychological problems among adults [50,51,52].

### Strengths and Limitations

Few studies have explored the prevalence of mental distress and social support among university students in the Middle East. To the best of our knowledge, this is the first study of its kind in the Middle East. The study has several strengths. Firstly, the study uses a large sample size (more than 1000 students were involved in this study). Secondly, our study addressed an important topic as mental distress has a negative, multidimensional impact on students’ health and performance. At the same time, this study has some limitations. The cross-sectional study design itself is not able to identify causality between the study variables. Due to the scarcity of studies on this topic in the Middle East, we encountered difficulties in comparing our findings with Arabic-speaking countries with a similar culture. However, we discussed our findings in comparison to other non-Arabic countries and highlighted the role of cultural variation. Finally, the use of an online survey for data collection could have missed some of the targeted population. However, this was common research practice during the pandemic as a previous study has documented that the use of online evaluation platforms during the COVID-19 pandemic increased significantly, specifically for education and research purposes [53].

## 5. Conclusions

Mental distress is prevalent among university students in Jordan. Further social support efforts are required by governments. Social support efforts should be directed towards high-risk populations (including females, students of non-medical colleges, students in their last years of study, those who have reported a history of chronic disease and those with a history of COVID-19 infection). Social support efforts could include self-help measures and professional mental health services as part of student health services that would be helpful to reduce the burden of mental distress of students and promote the mission of the integration of mental health in all university policies. Provided social support programs should be multidimensional (emotional, instrumental and informational).

## Figures and Tables

**Table 1:** Demographic characteristics of the study participants.

Demographic Variable	Frequency (%)
Age	
18–20 years	335 (31.5)
21–23 years	418 (39.3)
24–26 years	181 (17.0)
27–29 years	49 (4.6)
30 years and over	80 (7.5)
Gender	
Females	753 (70.8)
Males	310 (29.2)
Field of study	
Medical college	732 (68.9)
Other college	331 (31.1)
Year of study	
First year	145 (13.6)
Second year	153 (14.4)
Third year	222 (20.9)
Fourth year	204 (19.2)
Fifth year	206 (19.4)
Sixth year (for medicine, dentistry and PharmD)	7 (0.7)
Postgraduate study	126 (11.9)
Marital status	
Single	957 (90.0)
Married	95 (8.9)
Divorced	10 (0.9)
Widowed	1 (0.1)
Income level	
Less than 500 JD	702 (66.0)
500 to 1000 JD	237 (22.3)
1000 to 1500 JD	64 (6.0)
More than 1500 JD	60 (5.6)
Do you have any chronic condition?	
Yes	66 (6.2)
Do you have history of COVID-19 infection?	
Yes	386 (36.3)

**Table 2:** Mental distress and social support scores of the participants.

Mental Distress		
	Mean total score (SD)	
Total mental distress score	9.8 (5.5)	
Social support		
	Mean total score (SD)	Mean score per item (SD)
Significant other	13.9 (4.4)	3.5 (1.1)
Family	14.8 (3.9)	3.7 (1.0)
Friend	13.2 (4.2)	3.3 (1.1)
Total social support score	41.9 (10.3)	3.5 (0.9)

**Table 3:** Mean mental distress and social support score of university students stratified by demographic characteristics.

Demographic Variable	Mean Mental Distress Score (SD)	*p*-Value	Mean Social Support Score (SD)	*p*-Value
Age				
18–20 years	11.2 (5.5)	0.000 ***	40.6 (10.2)	0.041 *
21–23 years	9.5 (5.5)		42.2 (10.1)	
24–26 years	9.2 (5.6)		42.4 (10.2)	
27–29 years	8.9 (5.4)		42.1 (12.2)	
30 years and over	7.6 (4.7)		44.4 (10.2)	
Gender				
Males	7.6 (5.2)	0.000 ***	41.5 (9.7)	0.400
Females	10.7 (5.4)		42.1 (10.5)	
Field of study				
Medical college	9.4 (5.5)	0.001 **	42.3 (9.9)	0.046 *
Other college	10.7 (5.4)		40.9 (11.0)	
Year of study				
First year	10.6 (5.3)	0.000 ***	40.6 (10.8)	0.001 **
Second year	11.1 (5.4)		40.8 (10.3)	
Third year	10.9 (5.7)		40.1 (10.1)	
Fourth year	9.3 (5.4)		44.0 (10.3)	
Fifth year	8.1 (5.4)		43.2 (9.6)	
Sixth year (for medicine, dentistry and PharmD)	7.3 (5.9)		41.7 (10.7)	
Postgraduate study	9.2 (5.3)		42.3 (10.4)	
Marital status				
Single	10.0 (5.7)	0.003 **	41.5 (10.4)	0.000 ***
Married	7.9 (5.0)		45.9 (7.9)	
Divorced	8.8 (4.7)		44.7 (10.8)	
Widowed	16.0		16.0	
Income level				
Less than 500 JD	10.1 (5.5)	0.033 *	41.1 (10.2)	0.005 **
500 to 1000 JD	8.9 (5.8)		43.9 (9.8)	
1000 to 1500 JD	9.5 (5.4)		43.1 (11.2)	
More than 1500 JD	10.1 (5.2)		41.9 (10.3)	
Do you have any chronic condition?				
No	9.7 (5.6)	0.031 *	42.1 (10.1)	0.078
Yes	11.3 (5.1)		39.7 (12.6)	
Do you have history of COVID-19 infection?				
No	9.6 (5.6)	0.082	41.3 (10.5)	0.010 *
Yes	10.2 (5.5)		43.0 (9.7)	

* *p* < 0.05; ** *p* < 0.01; *** *p* < 0.001.

**Table 4:** Binary logistic regression analysis.

Demographic Variable	Odds Ratio (95% CI)
Age	
18–20 years (Reference category)	1.0
21–23 years	0.9 (0.7–1.1)
24–26 years	0.7 (0.5–0.9) *
27–29 years	0.8 (0.5–1.5)
30 years and over	0.6 (0.4–1.0) *
Gender	
Males (Reference category)	1.0
Females	2.4 (1.8–3.1) ***
Field of study	
Medical college (Reference category)	1.0
Other college	1.6 (1.2–2.1) **
Year of study	
First year (Reference category)	1.0
Second year	1.5 (1.0–2.2) *
Third year	1.6 (1.1–2.2) **
Fourth year	0.8 (0.6–1.1)
Fifth year	0.5 (0.3–0.6) ***
Sixth year (for medicine, dentistry and PharmD)	0.7 (0.2–3.1)
Postgraduate study	0.9 (0.6–1.3)
Marital status	
Single (Reference category)	1.0
Married	0.5 (0.3–0.8) **
Divorced	1.2 (0.3–4.8)
Widowed	-
Income level	
Less than 500 JD (Reference category)	1.0
500 to 1000 JD	0.7 (0.5–0.9) *
1000 to 1500 JD	0.8 (0.4–1.3)
More than 1500 JD	1.1 (0.7–2.0)
Do you have any chronic condition?	
No (Reference category)	1.0
Yes	1.8 (1.0–3.3) *
Do you have history of COVID-19 infection?	
No (Reference category)	1.0
Yes	1.4 (1.1–1.8) *

* *p* < 0.05; ** *p* < 0.01; *** *p* < 0.001.

## Data Availability

Not applicable.
